# Mistletoe Infection in an Oak Forest Is Influenced by Competition and Host Size

**DOI:** 10.1371/journal.pone.0127055

**Published:** 2015-05-18

**Authors:** Radim Matula, Martin Svátek, Marcela Pálková, Daniel Volařík, Tomáš Vrška

**Affiliations:** 1 Department of Forest botany, Dendrology and Geobiocoenology, Faculty of Forestry and Wood Technology, Mendel University in Brno, Brno, Czech Republic; 2 Department of Silviculture, Faculty of Forestry and Wood Technology, Mendel University in Brno, Brno, Czech Republic; 3 Silva Tarouca Research Institute, Department of Forest Ecology, Brno, Czech Republic; Chinese Academy of Sciences, CHINA

## Abstract

Host size and distance from an infected plant have been previously found to affect mistletoe occurrence in woody vegetation but the effect of host plant competition on mistletoe infection has not been empirically tested. For an individual tree, increasing competition from neighbouring trees decreases its resource availability, and resource availability is also known to affect the establishment of mistletoes on host trees. Therefore, competition is likely to affect mistletoe infection but evidence for such a mechanism is lacking. Based on this, we hypothesised that the probability of occurrence as well as the abundance of mistletoes on a tree would increase not only with increasing host size and decreasing distance from an infected tree but also with decreasing competition by neighbouring trees. Our hypothesis was tested using generalized linear models (GLMs) with data on Loranthus europaeus Jacq., one of the two most common mistletoes in Europe, on 1015 potential host stems collected in a large fully mapped plot in the Czech Republic. Because many trees were multi-stemmed, we ran the analyses for both individual stems and whole trees. We found that the probability of mistletoe occurrence on individual stems was affected mostly by stem size, whereas competition had the most important effects on the probability of mistletoe occurrence on whole trees as well as on mistletoe abundance. Therefore, we confirmed our hypothesis that competition among trees has a negative effect on mistletoe occurrence.

## Introduction

Mistletoes are widespread aerial hemiparasites of woody plants, ranging from the boreal zone to the tropics [1]. They play an important role in maintaining forest diversity by providing keystone food resources for many animal species due to the availability of their fruit in seasons when other food is scarce [2]. However, certain mistletoe species are considered tree pests in forests and plantations [3–6] because they disturb the water and nutrient balances and reduce photosynthesis and respiration, thus debilitating infected trees [7–9]. Due to the debilitation, severe mistletoe infection may lead to serious damage of infected trees or even tree death [10–14].

The distribution of mistletoe plants in woody vegetation is usually spatially non-random. This pattern has been explained by several factors. The studied species, *Loranthus europaeus* Jacq., like all 940 species of the family *Loranthaceae*, is characterised by direct seed dispersal by birds [8], whose behaviour usually results in an aggregated spatial distribution of infected trees [9, 15]. This aggregation indicates that trees within a certain distance of an infected tree are more likely to be infected than trees located further away [6, 16–18]. However, the distance at which an infected tree affects the probability of infection of neighbouring trees in the forest is poorly known.

The occurrence of mistletoes on host trees has also been found to be correlated with individual tree characteristics such as tree size, biomass and the occurrence of branches of certain diameters [16, 19–21]. For this reason, the distribution of mistletoe plants in the forest is likely to coincide with the distribution of host trees with the most favourable tree characteristics.

In this study, we hypothesised that neighbourhood competition among host trees would be another important factor that affects mistletoe occurrence. In forests, competition for resources is one of the key factors that negatively influences tree growth and reproduction [22–25]. Competition also changes resource allocation within individual plants [26], implying that competition may also cause differences in the amount of resources in host tree tissues that are available for mistletoes. Watson [27] has suggested that parasitic plants are more likely to establish and grow on host plants with better access to resources. Therefore, we expected that increasing competition among trees would have a negative effect on mistletoe occurrence because trees that outcompete their neighbours usually have better access to resources and thus have more available resources for mistletoe than the trees that they suppress. In addition, for an individual tree, increasing neighbourhood competition is caused by increases in the number and/or size of neighbour trees, which decrease the probability that the tree will receive mistletoe seeds and thus decrease the probability that mistletoe will occur on the tree. Either by changes in resource availability for host trees or by changes in the probability of receiving mistletoe seeds, host tree competition is likely to have a significant effect on mistletoe occurrence, but empirical proof of this effect is lacking.

To test the effect of neighbourhood competition together with other two key factors, size and distance to an infected tree, on the occurrence of mistletoe, we used data on 1015 potential host oak tree stems of the mistletoe L. europaeus in a large fully mapped plot in the Czech Republic. Specifically, we tested the hypothesis that that the probability of occurrence as well as abundance of mistletoes on an individual tree would increase not only with increasing host size and decreasing distance from an infected tree but also with decreasing competition by neighbouring trees. In addition, by recording the exact positions of all measured trees, we were able to model the effect of the distance from the nearest infected tree on the probability of mistletoe infection and infection severity while controlling for the effects of other significant factors by which we were able to identify the range within which an infected tree affects neighbour trees and to what extent. By testing together host tree competition, tree size and the distance from nearest infected tree, we were also able to disentangle their individual effects on the probability of mistletoe occurrence as well as on infection severity, i.e., mistletoe abundance.

## Materials and Methods

### Study area

**Fig 1:**
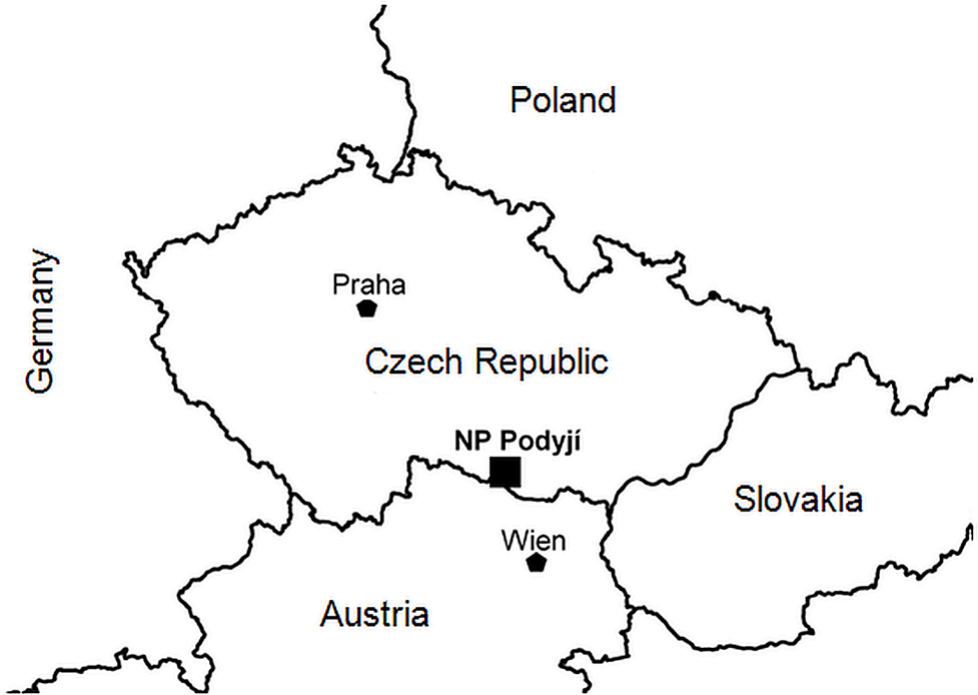
Location of the study site—Podyjí National Park (NP Podyjí).

The study was conducted in the area called Šobes in Podyjí National Park, which is located in the south of the Czech Republic on the border with Austria (bilateral Podyji/Thayatal National Park; Fig 1). For at least 500 years, forests in the Podyjí National Park were actively managed to provide firewood, and they were mostly maintained as coppices in which oak was preferred as a coppiced tree [28]. However, during the era of communism and the Cold War (1948–1989), the whole area was closed off because it was located in the military border zone with Austria. After the fall of communism in 1989, the area was kept as a protected area, and in 1991, it became Podyjí National Park [29]. The Podyjí National park Administration granted permission for the access to the area and for field data collection and it must be contacted for permission for any future research activities in the area (info@nppodyji.cz).

Because oak is the principal host species of *L*. *europaeus* in the Czech Republic [30], we established a 2.37 ha research plot, “Nad Šobesem”, which is situated in forest stands dominated by sessile oak (*Quercus petraea* (Matt) Liebl) (48°49'34'' N; 15°58'26'' E). Average tree density in the plot was 634 stems ha^-1^, average basal area 97.6 m^2^ ha^-1^, out of which sessile oak represented 96.5%. No other oak species, i.e. no other host species were present in the plot. The two species involved in our study were neither endangered nor protected. The plot was irregularly shaped because we wanted to cover the largest area of homogenous oak forest possible to capture as many trees infected with *L*. *europaeus* as possible. Because the plot was surrounded by closed forest, we did not consider any edge effect in our study. The forest stand in the research plot has a structure of one canopy layer high forest but many trees are multi-stemmed due to coppice origin. However, the forest stand has not been actively coppiced since the end of 19^th^ century and has become overmature (approximately 120 years) as a kind of transformation into high forest. The forest structure has been only slightly altered by selective spot cutting—singling out of some coppice stools in the 1980s-90s. (Pálková, unpublished data).

The stand was characterised as acidophilous oak woodlands. The mean annual temperature is 7.9°C, and the growing period length is 155–165 days. The long-term mean annual total precipitation is 460 mm (based on data from 1960–2010 from the nearest Znojmo weather station). The average slope of the plot was 3°. The environmental factors within the plot exhibited no apparent heterogeneity.

### Data collection

The positions of all standing tree stems with diameter at breast height (DBH) ≥ 7 cm were mapped using Field-Map technology (www.fieldmap.cz). Mapped stems were identified to species, and their DBH was measured using a diameter tape. The data set consisted of 1503 living stems.

Due to the coppice origin of the stand, many trees had more than one stem (i.e., formed polycormons). Therefore, by visually checking the basal part and upper roots for connections among stems, we assigned each stem to a multi-stemmed tree or, alternatively, to a single stem tree. In winter 2011/2012, we visually checked each oak stem for the presence of *L*. *europaeus* and, if present, *Loranthus* plants on the stem were counted. We used binoculars to enhance this visual checking, as some plants were relatively high up in the crown. The diameter of each mistletoe plant was also estimated (± 10 cm). Because all tree species in the studied plot were deciduous and lose leaves before winter, mistletoe plants were easily visible. In addition, *L*. *europaeus* is the only mistletoe that grows on sessile oak in central Europe, and thus there could be no confusion with other mistletoe species.

Using X/Y coordinates and the DBH of measured stems, we derived several variables. For trees with more than one stem (i.e., polycormons) we calculated the mean DBH of all stems (DBH_mean_) and the mean range of polycormons (Range) as the mean of two perpendicular polycormon diameters. As a simple measure of competition, we calculated the basal area of all stems (BA), the basal area of stems with DBH > 15 cm (BA15) and the basal area of stems with DBH > 30 cm (BA30) within a radius of 12 m around each stem. BA15 and BA30 were used to separate the effects of competition from all neighbouring stems from the effects of larger stems. Furthermore, as a more complex measure of competition, we calculated the tree-level density-dependent Hegyi competition index [31] for both stems (CI_stem_) and trees (CI_tree_), also within a radius of 12 m around each stem or tree. The formula of the Hegyi competition index is as follows:

$${\text{CI}}_{\text{Hegyi}}=\sum_{\text{j}=1}^{n}\frac{d_{j}}{d_{i}\left(l_{\text{ij}}+1\right)}$$

where d_i_ is the DBH of subject stem or BA of subject tree (for polycormons) i, d_j_ is the DBH of competitor stem or BA of tree j and l_ij_ is the distance between subject stem or tree i and competitor stem or tree j (DBH in cm, distance in m). To calculate the distance between multi-stemmed trees, we located their spatial centres (X/Y coordinates) as the points where the total distances to all stems within given multi-stemmed tree were the least possible. The Hegyi index increases with an increase in competition from neighbouring trees. Because of the radius, trees located < 12 m from the plot boundaries were removed from the statistical analyses. Also, broken trees were removed from the statistical analyses, as the absence of mistletoe could be simply due to the absence of some part of the tree crown. The exclusion of boundary and broken trees left 1015 target stems in total.

We also calculated the distance from each tree to the nearest infected stem (Distance). To separate the effects of trees with only small mistletoes from trees with larger mistletoes that may have a stronger effect on other trees, we also calculated the distance to the nearest tree with mistletoe greater than 50 cm in diameter (Distance50) and the distance to the nearest tree with mistletoe greater than 100 cm in diameter (Distance100). Distances as variables were calculated only for stems because they are likely to play an important role in the spread of seeds from one stem to another, which is likely to act at the stem level but not at the whole tree level of multi-stemmed trees.

### Data analysis

To quantify the effects of tree size, competition and position relative to infected trees on the probability of mistletoe infection of stems and trees (binary response variable: infected/not infected), we used generalized linear models (GLMs) with a binomial error distribution and logit link function. As explanatory variables for the probability of mistletoe infection of stems, we used DBH, Distance, Distance50, Distance100, BA, BA15, BA30 and CI_stem_. As explanatory variables for the probability of mistletoe infection of trees, we used mean DBH, CI_tree_ and Range. We used stepwise variable elimination from the maximal model as the model selection method using AICc [32] as a model criterion. Because CI_stem_ was correlated with DBH (Pearson’s r = 0.66) and CI_tree_ with Range (Pearson’s r = 0.68), we created models with each of the correlated variables separately and then compared the final models based on the difference in their AICcs (∆AICc). When ∆AICc was > 4, we chose the better model (i.e., the model with lower AICc) and considered the other models unimportant [32]. When ∆AICc was < 4, we calculated Akaike’s weights (w) for each model, which we then used as a measure of the importance of the explanatory variables in a given model [32]. In the same way, we tested the magnitudes of the effects of Distance, Distance50, and Distance100 and of BA, BA15, and BA30 because they were likely to be correlated. We first ran the analyses with each distance separately and then compared them by AICcs.

To quantify the effects of the tested explanatory variables on the infection severity expressed as number of mistletoes per stem, we used a GLM with a Poisson error distribution and a log link. The explanatory variables and the model selection methods were exactly the same as in the GLMs for probability of mistletoe infection of stems (paragraph above).

All analyses and calculations were performed in the R2.12.0 statistical environment [33]. The AICc and w calculations were made using the “*MuMIn*” package, and the “*ggplot2*” package [34] was used to create all presented figures.

## Results

### Host preference and competition

The infection rate of oak stems was 12.9% (131 of 1015 stems). Similarly, the whole tree infection rate was 14.8% (105 of 710 trees).

**Fig 2:**
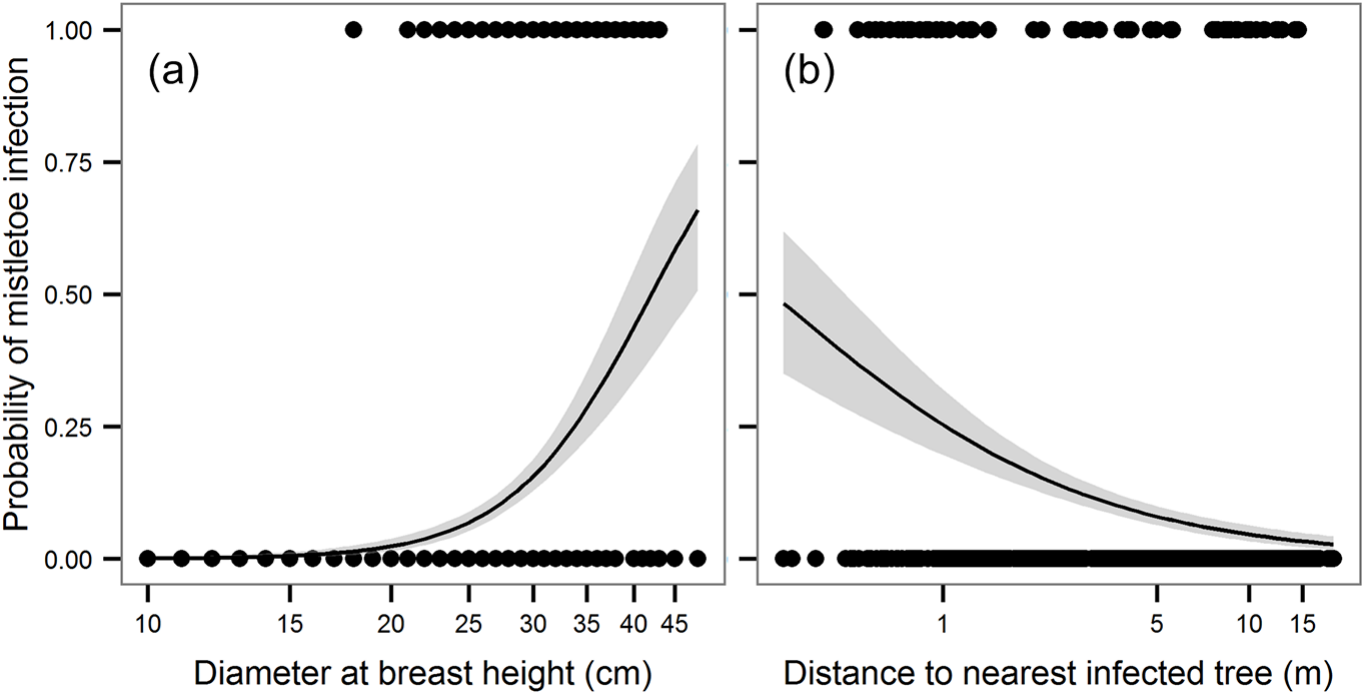
Probability of mistletoe infection of stems in relation to diameter at breast height a) and distance to the nearest infected tree b). The curves and 95% confidence intervals (shown in grey) represent partial effects derived from GLM with a binomial error distribution (link = logit).

The probability of mistletoe infection at the stem level significantly increased with increasing stem diameter but decreased with increasing distance from the nearest infected stem. The probability of infection was 0 for the smallest stems (< 16 cm), and from stem diameter 16 to approx. 26 cm it increased slowly, then rose steeply from 27 cm (Fig 2). The probability of infection of oak stems strongly decreased with increasing distance from an infected stem from 0–5 m, but for longer distances, the effect of distance to the nearest infected tree was much smaller (Fig 2). Distance50 and Distance100 were significantly poorer predictors than Distance from infected tree without differentiating mistletoe size (∆AICc >11.3; Table 1). Models using stem competition index instead of stem diameter were significant (P<0.05), but due to the much larger value of AICc of the best model with stem diameter (∆AICc >18.53; Table 1), they were considered unimportant. Neither of the stem basal area variables (BA, BA15, and BA30) affected the probability of infection.

**Table 1:** Four best GLMs with binomial error distributions for the probability of mistletoe (*Loranthus europaeus*) infection of individual stems as a response variable.

Intercept	CI_stem_	DBH	Distance	Distance50	Distance100	AICc	weight
0.0	-	4.9	-2.2	-	-	504.3	0.996
0.0	-	4.9	-	-	-1.8	515.6	0.004
0.0	-4.8	-	-2.5	-	-	522.9	0
0.0	-	4.8	-	-1.2	-	532.5	0

The zero mean and unit variance standarded coefficients, Akaike weight (weight) and AICc for each model are shown. CI_stem_ stands for Hegyi competition index for stems, DBH diameter at breast height, Distance means a distance from the nearest infected tree, Distance50 the distance to the nearest tree with mistletoe greater than 50 cm in diameter and Distance100 the distance to the nearest tree with mistletoe greater than 100 cm in diameter.

**Fig 3:**
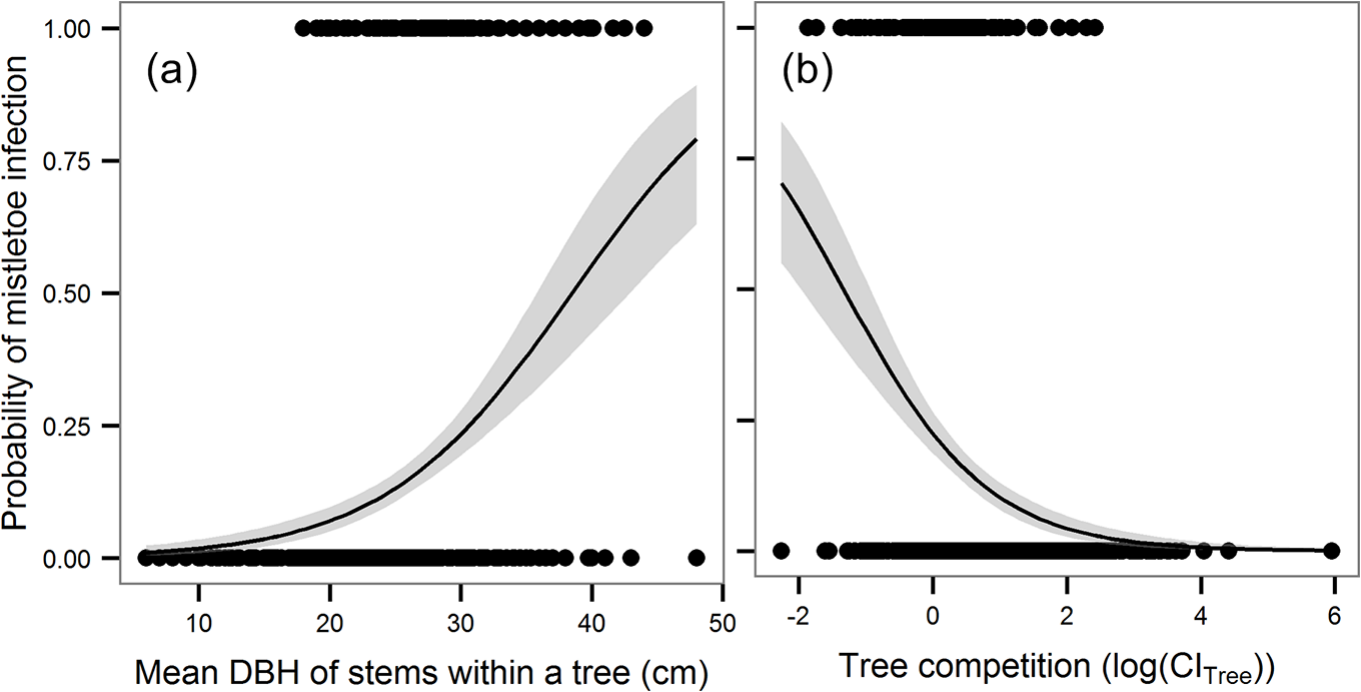
Probability of mistletoe infection of a tree in relation to mean diameter at breast height (DBH) a) and tree competition (CI_tree_) b). The curves and 95% confidence intervals (shown in grey) represent partial effects derived from GLM with a binomial error distribution (link = logit).

The probability of infection of whole trees increased significantly with an increase in mean stem diameter but decreased with an increase in tree competition index (Table 2). The increase in probability of infection with mean stem diameter was especially steep for values greater than 21 cm (Fig 3). The decrease in probability of infection with increasing competition was steep for lower values of the tree competition index but tended to stabilise at higher values of tree competition index (Fig 3). The range and basal area of whole trees had also significant positive effects on the probability of infection in trees, but the best model including those variables had w = 0.16 compared to w = 0.839 of the model with mean stem diameter and the tree competition index; and therefore, they were of much lower importance (Table 2).

**Table 2:** Four best GLMs with binomial error distributions for the probability of mistletoe (*Loranthus europaeus*) infection of whole trees as a response variable.

Intercept	Basal area	CI_tree_	DBH_tree_	Range	AICc	weight
0.0	-	-2.2	1.4	-	514.8	0.839
0.0	0.4	-	2.2	1.2	518.1	0.160
0.0	-	-2.8	-	-	531.5	0
0.0	2.8	-	-	-1.0	543.8	0

The zero mean and unit variance standardized coefficients, Akaike weight (weight) and AICc are shown for each model. Basal area represents total basal area of all stems per tree, CI_tree_ stands for Hegyi competition index for trees, DBH_tree_ mean DBH of all stems per tree and Range the mean polycormon range.

### Number of mistletoes per stem

**Fig 4:**
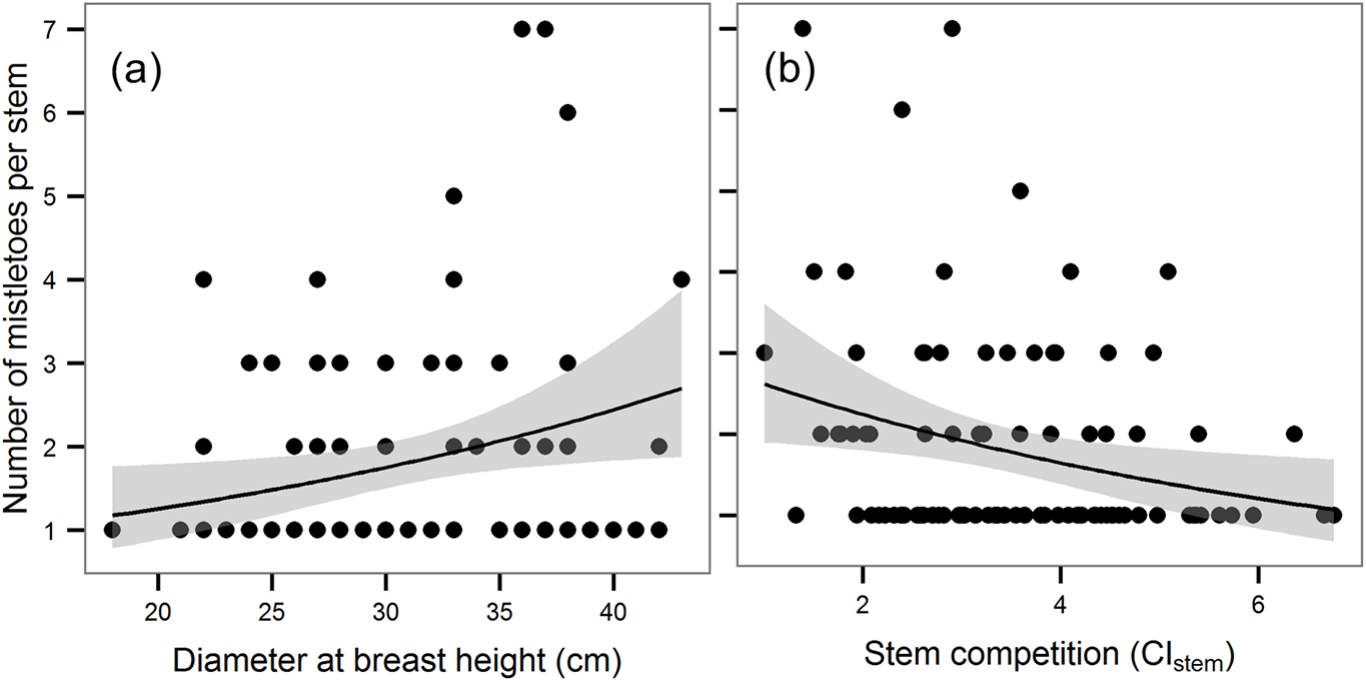
Number of mistletoe plants per tree in relation to diameter at breast height a) and stem competition (CI_stem_) b). The curves and 95% confidence intervals (shown in grey) represent partial effects derived from GLM with a Poisson error distribution (link = log).

The number of mistletoes per stem ranged from 1 to 7, but most of the infected stems had only one mistletoe (60%). The only variables that had a significant effect on the number of mistletoes per plant were CI_stem_ and DBH. The model with CI_stem_ as the only explanatory variable had the lowest value of AICc (295.4), followed closely by the model with only DBH (AICc = 296.3), but the CI_stem_ model had a much higher importance (w for model with CI_stem_ = 0.612, w for model with DBH = 0.388). The number of mistletoes significantly increased with decreasing DBH (Fig 4) and with increasing CI_stem_ (Fig 4). All other tested variables were left out of the models as insignificant (P > 0.05).

## Discussion

Our study showed that the probability of mistletoe infection of individual stems increases more significantly with stem size than with competition with neighbouring stems, whereas the probability of mistletoe infection of whole trees is significantly affected by both mean stem DBH and tree competition.

In general, competition among trees has a negative effect on resource availability for individual trees, which is reflected in a decline in growth with an increase in competition from neighbouring trees [24]. Therefore, trees facing greater competition from their neighbours are likely to contain less available resources for mistletoes in their tissues and thus are less likely to be parasitised by them than trees facing little or no neighbourhood competition. However, because the numbers of mistletoe seeds are usually limited, the decrease in the probability of mistletoe infection with an increase in competition from neighbouring trees may arise not from changes in resource availability but simply from a decreasing probability of receiving mistletoe seeds due to increased numbers of neighbour trees and/or due to increased shading by larger trees. Although we cannot directly reject any of the two possible mechanisms as a main driver of the negative mistletoe occurrence-tree competition relationship, our result that competition affects mistletoe occurrence only in the “whole” trees and not in individual stems supports the former mechanism. Unlike individual trees, stems within the same tree share the same root system and thus have similar resource availability, suggesting that, in our study, competition acted as a principal factor only in resource-variable trees but not in resource-similar stems. Therefore, the unimportant effect of competition among stems but the major effect of competition among trees on the probability of mistletoe infection supports the hypothesis that the effect of competition is primarily driven by changes in resource availability and not by changes in the probability of receiving mistletoe seeds.

Our finding that the probability of mistletoe infection increases with increasing tree size is in accordance with studies from other ecosystems [16, 20, 35, 36]. Aukema and Martínez del Rio [16] have found that within one host species, the tallest trees were more likely to be infected because they received more mistletoe seeds than smaller trees due to a higher number of bird visits, which is also likely to be a reason for the higher probability of infection of larger trees in our study. In addition, larger trees are also likely to have a greater surface area of the small twigs on which *L*. *europaeus* seeds are able to establish (twig diameter ≤ 6 cm; [30]).

We also found that increasing host tree competition as well as decreasing tree size lead to a decreasing number of mistletoe plants in infected trees but that the effect of competition is more important than the effect of tree size. Previous studies from other ecosystems [16, 36–38] have indicated similar effects of tree size, but neither of these studies tested the effect of competition. The reason for the decrease in the number of mistletoe plants with an increase in stem competition is most likely related to the behaviour of Mistle Thrushes (*Turdus viscivorus*), the most common bird species that feeds on mistletoe fruits and disperses mistletoe seeds [39] in Central Europe. These birds not only feed on mistletoe fruits but, from autumn to early spring, they also defend selected mistletoe-infected trees from other birds [40]. They preferentially select free-standing trees because they are easier to protect [41]. Therefore, in the forest, larger stems surrounded by few, relatively smaller stems have a higher probability that a Mistle Thrush will choose to defend and perch on them, which will, consequently, increase the number of mistletoe seeds arriving on such stems. Similarly, Monteiro et al. [38] have shown that the more frequent occurrence of mistletoes in taller trees was related to the behaviour of seed-dispersing birds, which preferred to perch on higher ground. Another explanation for the higher occurrence of mistletoes on taller stems might simply be that larger stems are older and, thus, have a longer time of exposure to infection by mistletoes, as suggested by Arruda et al. [37]. However, because most of the stems in our study plot originated from sprouts produced by stumps of trees that were harvested at the same time, which usually resprout within a year after harvest [42], the studied stems are most likely of similar age. Thus, the age of the stems is unlikely to have a significant effect on mistletoe occurrence in our case.

Our study also showed that the probability of infection increased in the vicinity of infected stems, but only at distances of less than 5 m. At such short distances, the crowns of neighbouring stems are likely to touch each other or even partially overlap and thus may act as one crown for birds. Therefore, when a crown of one of these stems is infected by chance by mistletoe that subsequently produces fruits, birds attracted by these fruits are likely to perch and thus disperse mistletoe seeds on the parent crown [15, 43, 44] as well as on the touching or overlapping neighbouring crowns. In addition, our finding that the distance to the nearest infected tree significantly affects the probability of infection but not the number of mistletoes per host indicates that a close infected tree significantly increases the probability of mistletoe seed arrival on an uninfected tree, but once it is infected, most seeds come from the mistletoe on the parent tree. These results corroborate Overton’s [18] finding that most mistletoe seeds are dispersed on the parent tree and its closest neighbours.

Our study showed that host competition, tree size and distance from infected tree play important role in determining the distribution of mistletoes in the forest. However, distance to an infected tree only affects the probability of mistletoe infection at short distances and does not affect infection severity, and thus appears to be less important in mistletoe infection than competition and size. Therefore, both tree competition and stem size can be considered as the principal determinants of mistletoe occurrence in the forest.
